# Transfusion-Dependent Anemia in a Simultaneous Pancreas and Kidney Transplant Recipient

**DOI:** 10.1155/2020/2841456

**Published:** 2020-04-08

**Authors:** Jeevan Prakash Gopal, David Taube, John Martin, Simona Deplano, Saral Desai, Vassilios Papalois, Anand Sivaprakash Rathnasamy Muthusamy

**Affiliations:** ^1^Imperial College Renal and Transplant Centre, Imperial College Healthcare NHS Trust, Hammersmith Hospital, London, UK; ^2^Department of Gastroenterology, Imperial College Healthcare NHS Trust, Charing Cross Hospital, London, UK; ^3^Department of Hematology, Imperial College Healthcare NHS Trust, Hammersmith Hospital, London, UK; ^4^Department of Pathology, Imperial College Healthcare NHS Trust, Hammersmith Hospital, London, UK; ^5^Department of Surgery and Cancer, Imperial College, London, UK

## Abstract

A case of transfusion-dependent anemia in a simultaneous pancreas and kidney (SPK) transplant recipient that masqueraded as gastrointestinal bleeding (GIB) is described. The anemia was attributed to bleeding from the donor duodenal cuff based on balloon enteroscopy findings. The patient underwent multiple contrast-enhanced computed tomography scans and multiple endoscopies with confounding features until, eventually, the diagnosis was established. We discuss the diagnostic difficulties and the therapeutic dilemma, along with the pitfalls in ascertaining the final diagnosis.

## 1. Introduction

Pancreas transplantation has been proven as the most effective treatment for selected patients with severe diabetic complications [1]. Better immunosuppression, routine use of antibody induction, advancement in organ preservation, and refinement in surgical technique have resulted in improved outcomes with 5- and 10-year graft function rates of 73% and 56%, respectively, for SPK transplantation [1].

## 2. Clinical Summary

A 45-year-old Asian man (cytomegalovirus (CMV) immunoglobulin (Ig)G positive) with poorly controlled type 2 diabetes mellitus and end-stage renal disease due to presumed diabetic nephropathy underwent a SPK transplantation from a 55-year-old four haplotype mismatched CMV seropositive deceased donor. The pancreas graft was implanted intraperitoneally with enteric exocrine drainage and systemic venous drainage into the recipient's inferior vena cava. Immunosuppression consisted of alemtuzumab induction and maintenance with tacrolimus, mycophenolate mofetil (MMF), and a short course of prednisolone (seven days). Postoperative recovery was uncomplicated with primary function of both the organs. He received intravenous immunoglobulin (IVIG) and additional steroids for presumed antibody-mediated rejection. Due to enhanced immunosuppression, prophylactic valganciclovir was continued for nine months.

Thirteen months after the transplant, he presented with an acute drop in haemoglobin (Hb) from 138 g/L to 86 g/L (normal 130-168) without any clinical evidence of bleeding. He was investigated with a contrast-enhanced computed tomography of the abdomen and pelvis (CTAP), an upper gastrointestinal endoscopy, a colonoscopy, and a faecal occult blood test, all of which were negative. He received two units of packed red blood cell (PRBC) transfusion and was discharged. He was readmitted two weeks later with recurrent haemoglobin drop from 112 g/L to 73 g/L associated with allograft dysfunction. He received further two units of PRBC transfusion, and the kidney was biopsied, which showed features of thrombotic microangiopathy. Serology for parvovirus B19 IgG and IgM were negative. CMV polymerase chain reaction (PCR) and BK virus PCR were negative. Hb improved to 103 g/L, and as the anemia was attributed to duodenal cuff bleeding due to pancreas allograft rejection, his tacrolimus dose was increased.

**Figure 1:**
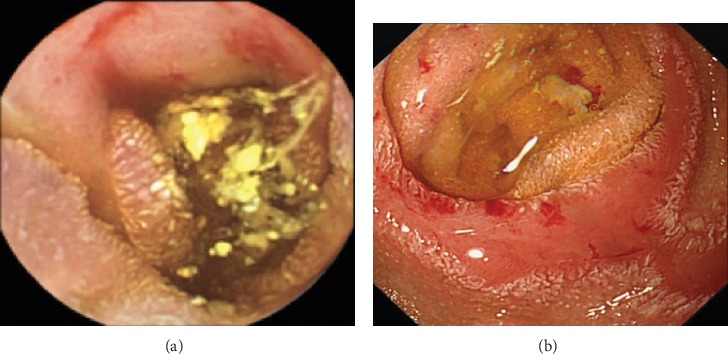
(a) Capsule endoscopy showing denuded mucosa with neovascularisation at the site of donor duodenal anastomosis. (b) Enteroscopic images showing ulceration at the donor duodenum with contact bleeding.

He then underwent a capsule endoscopy which showed denuded mucosa with neovascularisation at the site of donor duodenal anastomosis (Figure 1(a)). Thereafter, he underwent a balloon enteroscopy, which divulged ulceration near the donor duodenal anastomosis with contact bleeding from the adjacent donor duodenal cuff (Figure 1(b)). The bleeding points were addressed with argon plasma coagulation (APC). A biopsy of the transplant duodenum showed features of ischemic injury with ulceration; however, it was equivocal for rejection. A second external histology opinion was sought; this was reported as CMV duodenitis based on CMV inclusions in the biopsy. Treatment consisting of two weeks of intravenous ganciclovir followed by 450 mg valganciclovir and MMF cessation was instituted. A repeat CMV PCR was negative, and his Hb was stable at 93 g/L. Haematological investigations revealed absent reticulocytes at 0.0% (normal 0.45-1.82%) and normocytic anemia with polychromasia in peripheral blood film.

**Figure 2:**
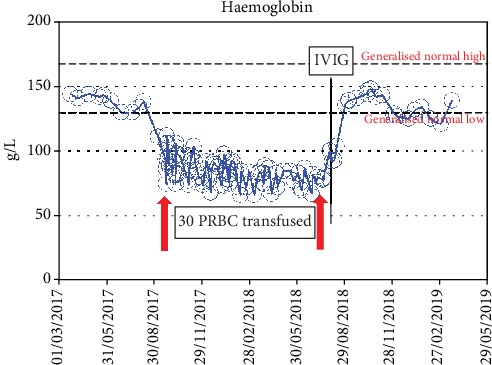
Haemoglobin trend before and after treatment is depicted along with the entire summary of blood transfusions.

He continued to be anemic, receiving fortnightly transfusions (thirty units of PRBC in total over nine months) (Figure 2). During a further visit, his Hb dropped again to 72 g/L. A repeat enteroscopy showed the same findings. Meanwhile, his pancreas graft was failing and was commenced on linagliptin 5 mg once daily. Given the context of a failing pancreas graft, continuing transfusion-dependent anemia, and enteroscopy findings of contact bleeding, graft pancreatectomy was considered a potential solution. On the other hand, as there was no overwhelming evidence of acute bleeding, a complete haematological workup was commenced.

**Figure 3:**
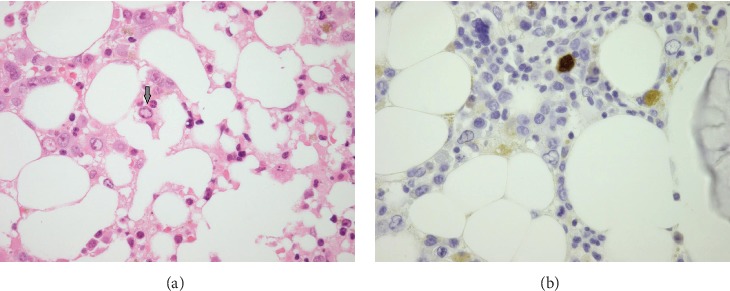
(a) Histological examination of a core biopsy specimen of the bone marrow showing intranuclear inclusions in erythroid precursor (arrow). (b) Immunophenotyping highlights intranuclear inclusions in erythroid precursor.

A bone marrow (BM) biopsy disclosed poorly formed erythroid islands with dyserythropoiesis and intranuclear inclusions in erythroid precursors (Figure 3(a)); immunophenotyping was positive for parvovirus (Figure 3(b)) and dysplastic features. BM flow cytometry was inconclusive. Eventually, parvovirus B19 DNA (deoxyribonucleic acid) PCR revealed 962 million copies/mL. A final diagnosis of parvovirus B19-induced pure red cell aplasia (PRCA) was established, and he was treated with two doses of IVIG 1 g/kg body weight (100 g). Anemia resolved rapidly, and Hb improved to 136 g/L within two weeks of IVIG. A repeat serology for parvovirus IgG was positive confirming seroconversion after a primary infection.

## 3. Discussion

Parvovirus B19-related pure red cell aplasia (PRCA) is rarely reported in SPK transplant recipients. We are reporting the third case of parvovirus B19 infection in SPK transplantation. The exact incidence of parvovirus B19 disease after transplantation is unknown but varies between 0 and 58% [2].

Parvovirus B19 is a nonenveloped, icosahedral, single-stranded DNA virus, belonging to the family Parvoviridae. Up to 80% of adults have antibodies against parvovirus B19 from previous exposure and presumably retain immunity to reinfection [3]. The virus displays tropism to the haematopoietic system and replicates only in erythroid precursor cells, due to the unique distribution of its cellular receptor, the red cell surface P antigen. The viral replication depends on mitotically active cells, and susceptibility to infection increases in erythroid precursors [2].

Parvovirus can cause a wide spectrum of diseases depending on host immunity. Immunocompetent individuals develop an asymptomatic infection or a mild self-limited illness. Immunocompromised patients, commonly present with anemia secondary to PRCA, are characterised by absent reticulocytes in the peripheral blood and absence of erythroblasts in the bone marrow, whilst other haematopoietic lineages are usually unaffected [2]. The parvovirus has also been associated with collapsing glomerulopathy and thrombotic microangiopathy leading to kidney allograft dysfunction [4]. The median time to onset of disease is seven weeks after transplantation, and most cases reported one year after transplantation are due to persistent viremia [4].

In immunocompetent patients, the enzyme-linked immunosorbent assay (ELISA) for IgG and IgM antibodies or PCR detection of the viral DNA from peripheral blood is diagnostic. The virus can also be identified by electron microscopy from bone marrow aspirate or biopsy samples. In immunocompromised patients, serology can be negative and PCR in peripheral blood has the best diagnostic accuracy [2]. BM examination should be performed if parvovirus is strongly suspected and serology and PCR are negative [2]. In addition, in situ hybridisation or immunohistochemistry can be done [2]. BM examination shows a characteristic morphologic feature of giant pronormoblasts with intranuclear inclusions along with paucity of mature erythroid precursors [2].

Establishing diagnosis in an immunocompromised patient is challenging, with dubious serological response adding to the conundrum. Serology was negative in our patient reported here. The virus was suspected on bone marrow examination and then confirmed by PCR. The resultant delay in diagnosis along with the spurious attribution of anemia to the bleeding source resulted in our patient undergoing an extensive diagnostic workup and multiple blood transfusions with secondary iron overload. On the contrary, elaborate investigation is justified as GIB due to arterial complications after pancreas transplantation (pseudoaneurysms and arterioenteric fistulas) is often missed in endoluminal imaging [5].

IVIG forms the treatment of choice for parvovirus infections, but there is a wide variation of practice in terms of dosing and duration of therapy [2]. IVIG typically contains a high titre of parvovirus B19-specific IgG as majority of the adult population have been previously exposed to parvovirus [6]. Due to high levels of clotting factor XI, IVIG has procoagulant activity [7], and thrombotic events after IVIG administration have been reported, typically occurring within the first day of receiving the first dose [8]. Anticoagulation dose escalation in conjunction with IVIG administration is crucial to prevent graft thrombosis. Other treatment options include modifying immunosuppression and conservative management. Spontaneous resolution of anemia has also been reported [2].

Persistently positive PCR after therapy is common, and it may take several months for the PCR to become negative [2]. Despite negative PCR after therapy, parvovirus may still remain in the bone marrow with a risk for recurrence. Recurrence is treated similar to primary infection. The role of human parvovirus B19 vaccine needs further appraisal.

This case report places emphasis on certain vital issues in the management of haematological complications after SPK transplantation:

- Endoscopic finding of bleeding can occur in the absence of a real intraluminal source for GIB
- High index of suspicion for an infectious cause of anemia has to be maintained in transplant recipients when the cause is obscure, especially with enhanced immunosuppression
- Graft pancreatectomy is a high-risk procedure with potential for enteric leak, pancreatic fistula, or life-threatening bleeding. Advising a graft pancreatectomy with a functioning graft should be reserved only when there is clear evidence of bleeding from the pancreas graft after all other causes have been excluded

## 4. Conclusion

Parvovirus B19 is an unusual cause for bone marrow suppression. A high index of suspicion is essential in an immunosuppressed patient. Molecular studies such as PCR for direct viral detection are diagnostic as serological response is unreliable. Swift treatment with a short course of IVIG usually results in successful clinical response.

## Abbreviations

g/L: Grams per liter
mL: Milliliter
kg: Kilogram.

## References

[1] Gruessner A. C., Gruessner R. W. (2016). Long-term outcome after pancreas transplantation: a registry analysis. *Current Opinion in Organ Transplantation*.

[2] Eid A. J., Ardura M. I., the AST Infectious Diseases Community of Practice (2019). Human parvovirus B19 in solid organ transplantation: guidelines from the American Society of Transplantation Infectious Diseases Community of Practice. *Clinical Transplantation*.

[3] Eid A. J., Posfay-Barbe K. M., the AST Infectious Diseases Community of Practice (2009). Parvovirus B19 in solid organ transplant recipients. *American Journal of Transplantation*.

[4] Eid A. J., Brown R. A., Patel R., Razonable R. R. (2006). Parvovirus B19 infection after transplantation: a review of 98 cases. *Clinical Infectious Diseases*.

[5] Yadav K., Young S., Finger E. B. (2017). Significant arterial complications after pancreas transplantation—a single-center experience and review of literature. *Clinical Transplantation*.

[6] Modrof J., Berting A., Tille B. (2008). Neutralization of human parvovirus B19 by plasma and intravenous immunoglobulins. *Transfusion*.

[7] Muthusamy A. S. R., Vaidya A. C., Sinha S. (2009). Pancreas allograft thrombosis following intravenous immunoglobulin administration to treat parvovirus B19 infection. *Transplant Infectious Disease*.

[8] Paran D., Herishanu Y., Elkayam O., Shopin L., Ben-Ami R. (2005). Venous and arterial thrombosis following administration of intravenous immunoglobulins. *Blood Coagulation & Fibrinolysis*.

